# Crystal structure and Hirshfeld surface analysis of 1-(4-bromo­phen­yl)-2-{[5-(pyridin-3-yl)-1,3,4-oxa­diazol-2-yl]sulfan­yl}ethan-1-one

**DOI:** 10.1107/S2056989017004819

**Published:** 2017-03-31

**Authors:** Huma Bano, Shafqat Hussain, Khalid M. Khan, Shahnaz Perveen, Sammer Yousuf

**Affiliations:** a H. E. J. Research Institute of Chemistry, International Center for Chemical and Biological Sciences, University of Karachi, Karachi-75270, Pakistan; b Karakoram International University, Gilgit, Pakistan; c PCSIR Laboratories Complex, Karachi, Pakistan; d H. E. J. Research Institute of Chemistry, International Center for Chemical and Biological Sciences, Sahrah-e-Dr. Salimuzzaman Siddiqui, Karachi-75280, Pakistan

**Keywords:** oxadizole, bromo­phen­yl, X-ray structure, Hirshfeld surface analysis, crystal structure

## Abstract

In the title compound, C_15_H_10_BrN_3_O_2_S, the dihedral angles between the 1,3,4-oxa­diazole ring and the 3-pyridinyl and bromo­benzene rings are 12.17 (15) and 18.74 (15)°, respectively. In the crystal, the mol­ecules are linked into [100] chains by way of C—H⋯O, C—H⋯N, C—H⋯S hydrogen bonds. The Hirshfeld surface analysis indicates that the most important contributions to the packing are H⋯H (19.5%), N⋯H (17.3%), C⋯H (15.5%), Br⋯H (11.7%), and O⋯H (11.0%) inter­actions.

## Abstract 2

In the crystal, the mol­ecules are linked into [100] chains by way of C—H⋯O, C—H⋯N, C—H⋯S hydrogen bonds. The Hirshfeld surface analysis indicates that the most important contributions to the packing are H⋯H (19.5%), N⋯H (17.3%), C⋯H (15.5%), Br⋯H (11.7%), and O⋯H (11.0%) inter­actions.

## Chemical context   

Substituted 1,3,4-oxa­diazo­les exhibit numerous biological activities such as anti­bacterial and anti­fungal (Prakash *et al.*, 2010 ▸, Chandrakantha *et al.*, 2010 ▸), anti­cancer (Abu-Zaied *et al.*, 2011 ▸), anti-inflammatory, analgesic (Husain *et al.*, 2009 ▸, Omar *et al.*, 1996 ▸), anti­convulsant and neurotoxic activities (Rajak *et al.*, 2010 ▸, Zarghi *et al.*, 2005 ▸). Chemical compounds having a 1,3,4-oxa­diazole moiety are also important contrib­utors towards the synthesis of biologically active heterocyclic compounds having anti­bacterial activity against resistant strains (Bharti *et al.*, 2010 ▸). As part of our studies in this area, we now describe the synthesis and structure of the title compound (I), a product of the condensation reaction between alcoholic solutions of 5-(3-pyrid­yl)-1,3,4-oxa­diazole-2-thiol and 2,4-di­bromo­aceto­phenone in the presence triethyl amine (Kashtoh *et al.*, 2014 ▸).
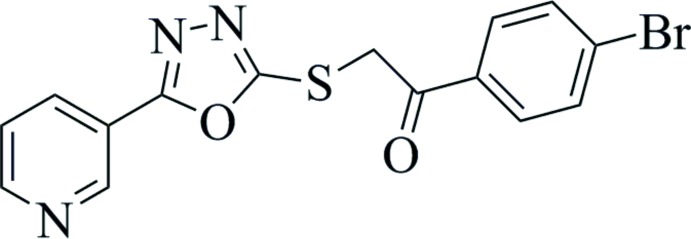


## Structural commentary   

**Figure 1:**
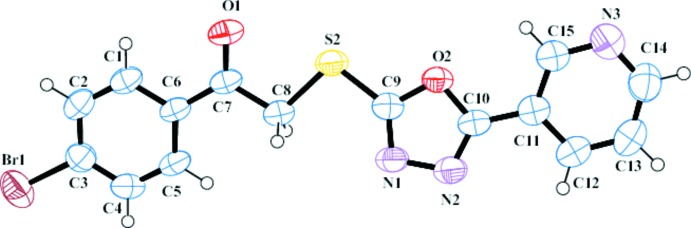
The mol­ecular structure of (I) with displacement ellipsoids drawn at the 30% probability level.

The structure of (I) (Fig. 1 ▸) is composed of three near-planar aromatic rings [bromo­phenyl (*A*), 3-pyridinyl (*B*) and 1,3,4-oxa­diazol (*C*)]. The inter-ring dihedral angles are *A*/*B* = 6.93 (15), *A*/*C* = 18.74 (15) and *B*/*C* = 12.17 (15)°. The C7—C8—S2—C9 torsion angle of 172.56 (17)° indicates approximate coplanarity of these atoms. Otherwise, geometrical data for (I) are similar to those found in structurally related compounds (Xia *et al.*, 2011 ▸; Xu *et al.*, 2005 ▸).

## Hydrogen bonding and Hirshfeld surface analysis   

**Figure 2:**
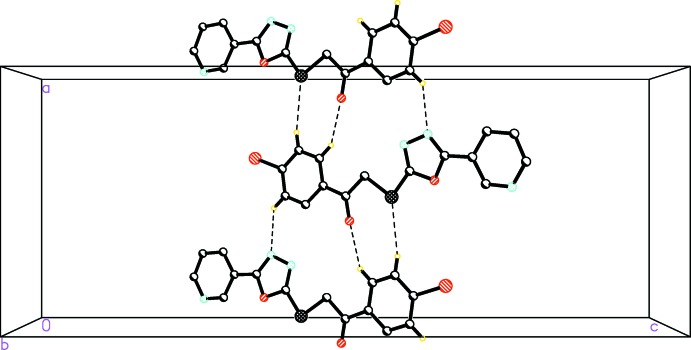
The crystal packing of the title compound (I). Only hydrogen atoms involved in hydrogen bonding (dashed lines) are shown.

**Figure 3:**
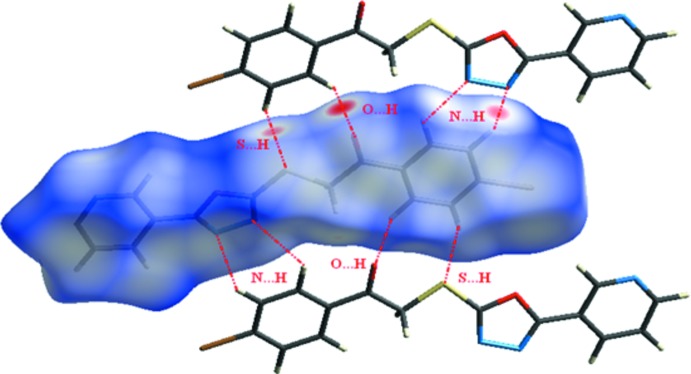
*d*_norm_ mapped on the Hirshfeld surface illustrating the inter­molecular contacts of the title compound. Dotted lines indicate hydrogen bonds.

**Figure 4:**
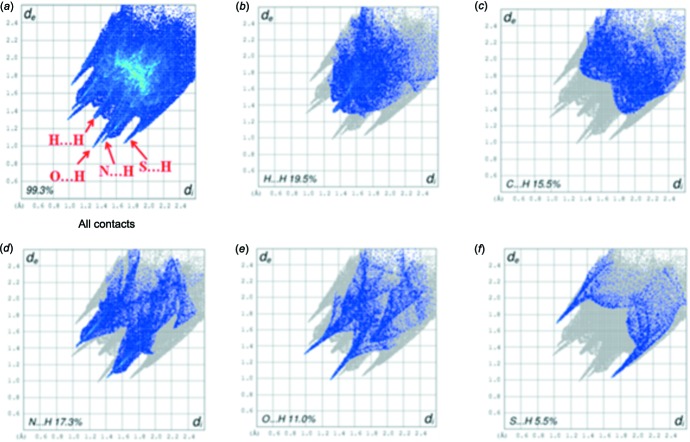
Fingerprint plots of the title compound, for (*a*) all, (*b*) H⋯H, (*c*) C⋯H, (*d*) N⋯H, (*e*) O⋯H and (*f*) S⋯H contacts. The outline of the full fingerprint plot is shown in grey. *d*_i_ is the closet inter­nal distance from a given point on the Hirshfeld surface and *d*_e_ is the closest external contact.

The packing of (I) is consolidated by C1—H1*B*⋯O1, C1—H2*B*⋯S2 and C4—H4*A*⋯N2 hydrogen bonds, which form chains running along *a*-axis direction (Fig. 2 ▸, Table 1 ▸). The Hirshfeld surface analysis (Hirshfeld, 1977 ▸) of the crystal structure indicates that the contribution of the H⋯H inter­molecular inter­actions to the crystal packing amounts to 19.5%, N⋯H = 17.3%, Br⋯H = 11.7% and O⋯H = 11.0%. Minor inter­molecular contacts for the cohesion of the structure are: C⋯O = 4.7%, C⋯C = 3.6% and others (Br⋯C, C⋯S, C⋯N, Br⋯S, N⋯N, Br⋯N, O⋯N)= 10.4%. These contacts are represented by conventional mapping of *d*_norm_ on the mol­ecular Hirshfeld surface, as shown in Fig. 3 ▸. The H⋯H contribution to the crystal packing is shown as a Hirshfeld surface two-dimensional fingerprint plot with red dots (Wolff *et al.*, 2012 ▸). The *d*_e_ (*y* axis) and *d*_i_ (*x* axis) values are the closest external and inter­nal distances (Å) from given points on the Hirshfeld surface (Fig. 4 ▸).

## Comparison with reported literature   

A database search disclosed a long list of compounds containing the 1,3,4-oxa­diazole moiety; however, only two examples of sulfanyl­ethanone-substituted 1,3,4-oxa­diazole derivatives were found, *viz*. 1,3-*bis*{[5-(pyridin-2-yl)-1,3,4-oxa­diazol-2-yl]sulfan­yl}propan-2-one (II) (Xia *et al.*, 2011 ▸) and 2-{5-[(1*H*-1,2,4-triazol-1-yl)-meth­yl]-1,3,4-oxa­diazol-2-yl­thio}-1-(2,4-di­chloro­phen­yl)ethanone (III) (Xu *et al.*, 2005 ▸). H⋯N inter­actions were found to be the most relevant inter­molecular inter­actions to form hydrogen bonds with neighboring mol­ecules. Therefore, *D*—H⋯N inter­actions were considered in a comparison with reported structures. In the crystal of (II), the mol­ecules are linked into a three-dimensional network *via* weak C—H⋯N hydrogen bonds (H⋯N distances = 2.51 and 2.54 Å) In (III), the C—H⋯N hydrogen bonds are found to be slightly weaker in comparison with the first structure (H⋯N distances = 2.41 Å). The change in substituents also changes the packing pattern towards zigzag chains extending along the *b*-axis direction. In addition, both (II) and (III) feature aromatic π–π stacking inter­actions, which are not observed in (I).

## Synthesis and crystallization   

5-(3-Pyrid­yl)-1,3,4-oxa­diazole-2-thiol (179 mg, 1 mmol) and triethyl amine (0.1 ml) were added in ethanol (10 ml) and stirred for 10 min in a round-bottomed flask. After 10 min, to the reaction mixture was slowly added 2 4-dibromaceto­phenone (278 mg, 1 mmol). The mixture was refluxed until complete consumption of starting materials, the progress of reaction being monitored by TLC. After 2 h, the precipitate that had formed was separated, washed with ethanol and recrystallized from methanol solution to afford colourless blocks (346 mg, 92% yield).

## Refinement   

Crystal data, data collection and structure refinement details are summarized in Table 2 ▸. H atoms were positioned geometrically with C—H = 0.93 Å (CH) or 0.97 Å (CH_2_) and constrained to ride on their parent atoms with *U*_iso_(H)= 1.2*U*_eq_(C).

## Supplementary Material

Crystal structure: contains datablock(s) global, I. DOI: 10.1107/S2056989017004819/hb7660sup1.cif

Structure factors: contains datablock(s) I. DOI: 10.1107/S2056989017004819/hb7660Isup2.hkl

Supporting information file. DOI: 10.1107/S2056989017004819/hb7660Isup3.cml

CCDC reference: 1540579

Additional supporting information:  crystallographic information; 3D view; checkCIF report

## Figures and Tables

**Table 1:** Hydrogen-bond geometry (Å, °)

*D*—H⋯*A*	*D*—H	H⋯*A*	*D*⋯*A*	*D*—H⋯*A*
C1—H1*B*⋯O1^i^	0.93	2.42	3.260 (3)	150
C2—H2*B*⋯S2^i^	0.93	2.86	3.716 (3)	153
C4—H4*A*⋯N2^ii^	0.93	2.58	3.372 (4)	144

Symmetry codes: (i) 
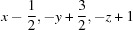
; (ii) 
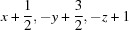
.

**Table 2:** Experimental details

Crystal data	
Chemical formula	C_15_H_10_BrN_3_O_2_S
*M*_r_	376.23
Crystal system, space group	Orthorhombic, *P* *b* *c* *a*
Temperature (K)	273
*a*, *b*, *c* (Å)	11.9144 (16), 8.3755 (12), 30.382 (4)
*V* (Å^3^)	3031.8 (7)
*Z*	8
Radiation type	Mo *K*α
μ (mm^−1^)	2.86
Crystal size (mm)	0.47 × 0.39 × 0.11

Data collection	
Diffractometer	Bruker SMART APEX CCD
Absorption correction	Multi-scan (*SADABS*; Bruker, 2000 ▸)
*T*_min_, *T*_max_	0.347, 0.746
No. of measured, independent and observed [*I* > 2σ(*I*)] reflections	16806, 2765, 2106
*R*_int_	0.038
(sin θ/λ)_max_ (Å^−1^)	0.606

Refinement	
*R*[*F* ^2^ > 2σ(*F* ^2^)], *wR*(*F* ^2^), *S*	0.035, 0.114, 1.13
No. of reflections	2765
No. of parameters	199
H-atom treatment	H atoms treated by a mixture of independent and constrained refinement
Δρ_max_, Δρ_min_ (e Å^−3^)	0.40, −0.25

Computer programs: *SMART* and *SAINT* (Bruker, 2000 ▸), *SHELXS97*, *SHELXL97* and *SHELXTL* (Sheldrick, 2008 ▸), *PARST* (Nardelli, 1995 ▸) and *PLATON* (Spek, 2009 ▸).

## supplementary crystallographic information

### Crystal data

**Table:**

C_15_H_10_BrN_3_O_2_S	*D*_x_ = 1.648 Mg m^−^^3^
*M**_r_* = 376.23	Mo *K*α radiation, λ = 0.71073 Å
Orthorhombic, *P**b**c**a*	Cell parameters from 3887 reflections
*a* = 11.9144 (16) Å	θ = 2.7–22.9°
*b* = 8.3755 (12) Å	µ = 2.86 mm^−^^1^
*c* = 30.382 (4) Å	*T* = 273 K
*V* = 3031.8 (7) Å^3^	Block, colorless
*Z* = 8	0.47 × 0.39 × 0.11 mm
*F*(000) = 1504	

### Data collection

**Table:**

Bruker SMART APEX CCD diffractometer	2765 independent reflections
Radiation source: fine-focus sealed tube	2106 reflections with *I* > 2σ(*I*)
Graphite monochromator	*R*_int_ = 0.038
ω scan	θ_max_ = 25.5°, θ_min_ = 1.3°
Absorption correction: multi-scan (SADABS; Bruker, 2000)	*h* = −14→13
*T*_min_ = 0.347, *T*_max_ = 0.746	*k* = −10→10
16806 measured reflections	*l* = −36→36

### Refinement

**Table:**

Refinement on *F*^2^	Primary atom site location: structure-invariant direct methods
Least-squares matrix: full	Secondary atom site location: difference Fourier map
*R*[*F*^2^ > 2σ(*F*^2^)] = 0.035	Hydrogen site location: inferred from neighbouring sites
*wR*(*F*^2^) = 0.114	H atoms treated by a mixture of independent and constrained refinement
*S* = 1.13	*w* = 1/[σ^2^(*F*_o_^2^) + (0.0639*P*)^2^ + 0.238*P*] where *P* = (*F*_o_^2^ + 2*F*_c_^2^)/3
2765 reflections	(Δ/σ)_max_ = 0.002
199 parameters	Δρ_max_ = 0.40 e Å^−^^3^
0 restraints	Δρ_min_ = −0.25 e Å^−^^3^

### Special details

**Table:**

Geometry. All esds (except the esd in the dihedral angle between two l.s. planes) are estimated using the full covariance matrix. The cell esds are taken into account individually in the estimation of esds in distances, angles and torsion angles; correlations between esds in cell parameters are only used when they are defined by crystal symmetry. An approximate (isotropic) treatment of cell esds is used for estimating esds involving l.s. planes.
Refinement. Refinement of F^2^ against ALL reflections. The weighted R-factor wR and goodness of fit S are based on F^2^, conventional R-factors R are based on F, with F set to zero for negative F^2^. The threshold expression of F^2^ > 2sigma(F^2^) is used only for calculating R-factors(gt) etc. and is not relevant to the choice of reflections for refinement. R-factors based on F^2^ are statistically about twice as large as those based on F, and R- factors based on ALL data will be even larger.

### Fractional atomic coordinates and isotropic or equivalent isotropic displacement parameters (Å^2^)

**Table:**

	*x*	*y*	*z*	*U*_iso_*/*U*_eq_	
Br1	0.32966 (4)	1.17413 (5)	0.651655 (12)	0.0855 (2)	
S2	0.48889 (6)	0.57198 (9)	0.42780 (3)	0.0573 (2)	
O1	0.58756 (16)	0.7313 (2)	0.49352 (7)	0.0627 (6)	
O2	0.42221 (15)	0.3878 (2)	0.36429 (6)	0.0519 (5)	
N1	0.2823 (2)	0.4393 (3)	0.40960 (8)	0.0593 (6)	
N2	0.2436 (2)	0.3373 (3)	0.37529 (9)	0.0632 (7)	
N3	0.4463 (3)	0.0865 (4)	0.25578 (10)	0.0847 (9)	
C1	0.3327 (2)	0.8827 (4)	0.54232 (11)	0.0561 (7)	
H1B	0.2798	0.8391	0.5233	0.067*	
C2	0.2986 (3)	0.9787 (4)	0.57656 (11)	0.0642 (8)	
H2B	0.2227	1.0005	0.5806	0.077*	
C3	0.3762 (3)	1.0418 (3)	0.60463 (9)	0.0561 (7)	
C4	0.4894 (2)	1.0117 (3)	0.59921 (10)	0.0572 (8)	
H4A	0.5416	1.0550	0.6186	0.069*	
C5	0.5238 (2)	0.9178 (3)	0.56513 (10)	0.0528 (7)	
H5A	0.5999	0.8979	0.5612	0.063*	
C6	0.4458 (2)	0.8510 (3)	0.53610 (9)	0.0450 (6)	
C7	0.4875 (2)	0.7497 (3)	0.49968 (9)	0.0468 (6)	
C8	0.4052 (2)	0.6676 (3)	0.46970 (10)	0.0495 (7)	
H8A	0.3543	0.7445	0.4566	0.059*	
H8B	0.3617	0.5892	0.4858	0.059*	
C9	0.3863 (2)	0.4642 (3)	0.40109 (9)	0.0501 (7)	
C10	0.3272 (2)	0.3106 (3)	0.35029 (10)	0.0514 (7)	
C11	0.3335 (2)	0.2139 (3)	0.31076 (11)	0.0541 (7)	
C12	0.2370 (3)	0.1585 (3)	0.29051 (11)	0.0621 (8)	
H12A	0.1665	0.1820	0.3020	0.075*	
C13	0.2476 (3)	0.0681 (4)	0.25307 (11)	0.0717 (10)	
H13A	0.1842	0.0287	0.2389	0.086*	
C14	0.3520 (3)	0.0365 (4)	0.23684 (12)	0.0779 (10)	
H14A	0.3575	−0.0232	0.2111	0.093*	
C15	0.4355 (3)	0.1747 (4)	0.29175 (12)	0.0702 (9)	
H15A	0.5005	0.2127	0.3051	0.084*	

### Atomic displacement parameters (Å^2^)

**Table:**

	*U*^11^	*U*^22^	*U*^33^	*U*^12^	*U*^13^	*U*^23^
Br1	0.1083 (4)	0.0886 (3)	0.0597 (3)	−0.00727 (19)	0.02208 (18)	−0.00144 (17)
S2	0.0409 (4)	0.0600 (4)	0.0711 (5)	−0.0033 (3)	0.0054 (3)	−0.0031 (4)
O1	0.0357 (14)	0.0716 (13)	0.0808 (15)	0.0013 (10)	−0.0025 (10)	0.0025 (11)
O2	0.0407 (11)	0.0572 (10)	0.0578 (12)	−0.0024 (9)	0.0065 (9)	0.0010 (9)
N1	0.0405 (14)	0.0658 (15)	0.0716 (17)	−0.0039 (11)	0.0083 (12)	−0.0052 (12)
N2	0.0454 (16)	0.0682 (16)	0.0759 (18)	−0.0064 (11)	0.0074 (14)	−0.0062 (13)
N3	0.075 (2)	0.103 (2)	0.076 (2)	0.0034 (17)	0.0039 (16)	−0.0197 (17)
C1	0.0354 (17)	0.0653 (17)	0.068 (2)	−0.0096 (13)	−0.0067 (13)	0.0019 (15)
C2	0.0432 (18)	0.075 (2)	0.074 (2)	−0.0041 (15)	0.0102 (15)	0.0014 (17)
C3	0.059 (2)	0.0566 (16)	0.0527 (17)	−0.0062 (14)	0.0036 (14)	0.0095 (13)
C4	0.055 (2)	0.0570 (17)	0.0592 (19)	−0.0102 (13)	−0.0151 (14)	0.0116 (14)
C5	0.0409 (16)	0.0536 (16)	0.0638 (18)	−0.0013 (12)	−0.0102 (13)	0.0114 (14)
C6	0.0343 (15)	0.0450 (13)	0.0558 (16)	−0.0031 (11)	−0.0059 (12)	0.0128 (12)
C7	0.0350 (18)	0.0455 (14)	0.0600 (17)	−0.0014 (11)	−0.0046 (12)	0.0130 (12)
C8	0.0381 (16)	0.0504 (15)	0.0601 (17)	−0.0002 (11)	0.0001 (12)	0.0039 (12)
C9	0.0451 (18)	0.0450 (14)	0.0600 (18)	0.0018 (12)	0.0039 (13)	0.0057 (13)
C10	0.0409 (18)	0.0515 (16)	0.0619 (19)	−0.0025 (12)	0.0015 (13)	0.0096 (13)
C11	0.053 (2)	0.0515 (15)	0.0580 (18)	0.0001 (12)	−0.0009 (13)	0.0075 (13)
C12	0.053 (2)	0.0605 (18)	0.073 (2)	−0.0053 (14)	−0.0073 (16)	0.0071 (15)
C13	0.074 (3)	0.069 (2)	0.072 (2)	−0.0099 (18)	−0.0188 (18)	−0.0001 (17)
C14	0.085 (3)	0.079 (2)	0.070 (2)	0.002 (2)	−0.010 (2)	−0.0093 (18)
C15	0.055 (2)	0.087 (2)	0.069 (2)	−0.0046 (16)	0.0017 (16)	−0.0092 (17)

### Geometric parameters (Å, º)

**Table:**

Br1—C3	1.891 (3)	C4—C5	1.363 (4)
S2—C9	1.722 (3)	C4—H4A	0.9300
S2—C8	1.804 (3)	C5—C6	1.398 (4)
O1—C7	1.217 (3)	C5—H5A	0.9300
O2—C9	1.357 (3)	C6—C7	1.480 (4)
O2—C10	1.372 (3)	C7—C8	1.504 (4)
N1—C9	1.283 (4)	C8—H8A	0.9700
N1—N2	1.424 (3)	C8—H8B	0.9700
N2—C10	1.272 (4)	C10—C11	1.451 (4)
N3—C15	1.326 (4)	C11—C12	1.384 (4)
N3—C14	1.330 (5)	C11—C15	1.385 (4)
C1—C2	1.376 (4)	C12—C13	1.372 (4)
C1—C6	1.387 (4)	C12—H12A	0.9300
C1—H1B	0.9300	C13—C14	1.364 (4)
C2—C3	1.365 (4)	C13—H13A	0.9300
C2—H2B	0.9300	C14—H14A	0.9300
C3—C4	1.382 (4)	C15—H15A	0.9300
			
C9—S2—C8	99.97 (13)	C7—C8—H8A	110.6
C9—O2—C10	102.6 (2)	S2—C8—H8A	110.6
C9—N1—N2	105.2 (2)	C7—C8—H8B	110.6
C10—N2—N1	106.8 (2)	S2—C8—H8B	110.6
C15—N3—C14	116.7 (3)	H8A—C8—H8B	108.7
C2—C1—C6	120.1 (3)	N1—C9—O2	113.2 (2)
C2—C1—H1B	119.9	N1—C9—S2	132.5 (2)
C6—C1—H1B	119.9	O2—C9—S2	114.32 (19)
C3—C2—C1	119.9 (3)	N2—C10—O2	112.2 (3)
C3—C2—H2B	120.0	N2—C10—C11	129.3 (3)
C1—C2—H2B	120.0	O2—C10—C11	118.5 (2)
C2—C3—C4	121.1 (3)	C12—C11—C15	117.7 (3)
C2—C3—Br1	120.0 (2)	C12—C11—C10	120.8 (3)
C4—C3—Br1	118.9 (2)	C15—C11—C10	121.5 (3)
C5—C4—C3	119.2 (3)	C13—C12—C11	118.5 (3)
C5—C4—H4A	120.4	C13—C12—H12A	120.8
C3—C4—H4A	120.4	C11—C12—H12A	120.8
C4—C5—C6	120.7 (3)	C14—C13—C12	119.4 (3)
C4—C5—H5A	119.6	C14—C13—H13A	120.3
C6—C5—H5A	119.6	C12—C13—H13A	120.3
C1—C6—C5	118.9 (3)	N3—C14—C13	123.6 (4)
C1—C6—C7	122.5 (3)	N3—C14—H14A	118.2
C5—C6—C7	118.6 (2)	C13—C14—H14A	118.2
O1—C7—C6	121.1 (2)	N3—C15—C11	124.1 (3)
O1—C7—C8	119.2 (3)	N3—C15—H15A	117.9
C6—C7—C8	119.7 (2)	C11—C15—H15A	117.9
C7—C8—S2	105.69 (19)		
			
C9—N1—N2—C10	0.6 (3)	C10—O2—C9—N1	0.1 (3)
C6—C1—C2—C3	−0.5 (5)	C10—O2—C9—S2	178.86 (18)
C1—C2—C3—C4	0.3 (5)	C8—S2—C9—N1	−7.1 (3)
C1—C2—C3—Br1	179.8 (2)	C8—S2—C9—O2	174.34 (19)
C2—C3—C4—C5	0.2 (4)	N1—N2—C10—O2	−0.6 (3)
Br1—C3—C4—C5	−179.3 (2)	N1—N2—C10—C11	179.7 (3)
C3—C4—C5—C6	−0.6 (4)	C9—O2—C10—N2	0.3 (3)
C2—C1—C6—C5	0.1 (4)	C9—O2—C10—C11	−179.9 (2)
C2—C1—C6—C7	−179.5 (3)	N2—C10—C11—C12	12.1 (5)
C4—C5—C6—C1	0.5 (4)	O2—C10—C11—C12	−167.6 (2)
C4—C5—C6—C7	−179.9 (2)	N2—C10—C11—C15	−168.4 (3)
C1—C6—C7—O1	175.7 (3)	O2—C10—C11—C15	11.9 (4)
C5—C6—C7—O1	−3.8 (4)	C15—C11—C12—C13	0.2 (4)
C1—C6—C7—C8	−4.4 (4)	C10—C11—C12—C13	179.7 (3)
C5—C6—C7—C8	176.0 (2)	C11—C12—C13—C14	−0.4 (5)
O1—C7—C8—S2	−4.8 (3)	C15—N3—C14—C13	−1.6 (6)
C6—C7—C8—S2	175.32 (19)	C12—C13—C14—N3	1.2 (6)
C9—S2—C8—C7	172.56 (17)	C14—N3—C15—C11	1.4 (5)
N2—N1—C9—O2	−0.4 (3)	C12—C11—C15—N3	−0.7 (5)
N2—N1—C9—S2	−178.9 (2)	C10—C11—C15—N3	179.8 (3)

### Hydrogen-bond geometry (Å, º)

**Table:**

*D*—H···*A*	*D*—H	H···*A*	*D*···*A*	*D*—H···*A*
C1—H1*B*···O1^i^	0.93	2.42	3.260 (3)	150
C2—H2*B*···S2^i^	0.93	2.86	3.716 (3)	153
C4—H4*A*···N2^ii^	0.93	2.58	3.372 (4)	144

Symmetry codes: (i) *x*−1/2, −*y*+3/2, −*z*+1; (ii) *x*+1/2, −*y*+3/2, −*z*+1.

## References

[bb1] Abu-Zaied, M., El-Telbani, E. M., Elgemeie, G. H. & Nawwar, G. A. (2011). *Eur. J. Med. Chem.* **46**, 229–235. 10.1016/j.ejmech.2010.11.008 21115211

[bb2] Bharti, S. K., Nath, G., Tilak, R. & Singh, S. K. (2010). *Eur. J. Med. Chem.* **45**, 651–660. 10.1016/j.ejmech.2009.11.008 19932927

[bb3] Bruker (2000). *SADABS*, *SMART* and *SAINT*. Bruker AXS Inc., Madison, Wisconsin, USA.

[bb4] Chandrakantha, B., Shetty, P., Nambiyar, V., Isloor, N. & Isloor, A. M. (2010). *Eur. J. Med. Chem.* **45**, 1206–1210. 10.1016/j.ejmech.2009.11.046 20004043

[bb5] Hirshfeld, H. L. (1977). *Theor. Chim. Acta*, **44**, 129–138.

[bb6] Husain, A., Ahmad, A., Alam, M. M., Ajmal, M. & Ahuja, P. (2009). *Eur. J. Med. Chem.* **44**, 3798–3804. 10.1016/j.ejmech.2009.04.009 19457595

[bb8] Kashtoh, H., Hussain, S., Khan, A., Saad, S. M., Khan, J. A. J., Khan, K. M., Perveen, S. & Choudhary, M. I. (2014). *Bioorg. Med. Chem.* **22**, 5454–5465. 10.1016/j.bmc.2014.07.032 25151088

[bb18] Nardelli, M. (1995). *J. Appl. Cryst.* **28**, 659.

[bb9] Omar, F. A., Mahfouz, N. M. & Rahman, M. A. (1996). *Eur. J. Med. Chem.* **31**, 819–825. 10.1016/0223-5234(96)83976-6 22026938

[bb10] Prakash, O., Kumar, M., Kumar, R., Sharma, C. & Aneja, K. R. (2010). *Eur. J. Med. Chem.* **45**, 4252–4257. 10.1016/j.ejmech.2010.06.023 20630627

[bb11] Rajak, H., Deshmukh, R., Veerasamy, R., Sharma, A. K., Mishra, P. & Kharya, M. D. (2010). *Bioorg. Med. Chem. Lett.* **20**, 4168–4172. 10.1016/j.bmcl.2010.05.059 20558061

[bb12] Sheldrick, G. M. (2008). *Acta Cryst.* A**64**, 112–122. 10.1107/S0108767307043930 18156677

[bb13] Spek, A. L. (2009). *Acta Cryst.* D**65**, 148–155. 10.1107/S090744490804362X PMC2631630 19171970

[bb14] Wolff, S. K., Grimwood, D. J., McKinnon, J. J., Turner, M. J., Jayatilaka, D. & Spackman, M. A. (2012). *Crystal Explorer*. University of Western Australia.

[bb15] Xia, C.-H., Mao, C.-B. & Wu, B.-L. (2011). *Acta Cryst.* E**67**, o413. 10.1107/S1600536811001140 PMC3051430 21523084

[bb16] Xu, L.-Z., Yu, G.-P., Yin, S.-M., Zhou, K. & Yang, S.-H. (2005). *Acta Cryst.* E**61**, o3375–o3376.

[bb17] Zarghi, A., Tabatabai, S. A., Faizi, M., Ahadian, A., Navabi, P., Zanganeh, V. & Shafiee, A. (2005). *Bioorg. Med. Chem. Lett.* **15**, 1863–1865. 10.1016/j.bmcl.2005.02.014 15780622

